# The Skeleton of the Staghorn Coral *Acropora millepora*: Molecular and Structural Characterization

**DOI:** 10.1371/journal.pone.0097454

**Published:** 2014-06-03

**Authors:** Paula Ramos-Silva, Jaap Kaandorp, Frédéric Herbst, Laurent Plasseraud, Gérard Alcaraz, Christine Stern, Marion Corneillat, Nathalie Guichard, Christophe Durlet, Gilles Luquet, Frédéric Marin

**Affiliations:** 1 UMR 6282 Biogéosciences, Université de Bourgogne, Dijon, France; 2 Section Computational Science, Faculty of Science, University of Amsterdam, Amsterdam, The Netherlands; 3 UMR 5209, Université de Bourgogne, Dijon, France; 4 UMR 6302, Institut de Chimie Moléculaire, Université de Bourgogne, Dijon, France; 5 UMR 1347, Agroécologie INRA, Université de Bourgogne, AgroSup Dijon, Pôle Mécanisme & Gestion Interactions Plantes Micro-organismes, ERL 6300, Dijon, France; 6 UMR 7245, Muséum National d'Histoire Naturelle, Paris, France; Pennsylvania State University, United States of America

## Abstract

The scleractinian coral *Acropora millepora* is one of the most studied species from the Great Barrier Reef. This species has been used to understand evolutionary, immune and developmental processes in cnidarians. It has also been subject of several ecological studies in order to elucidate reef responses to environmental changes such as temperature rise and ocean acidification (OA). In these contexts, several nucleic acid resources were made available. When combined to a recent proteomic analysis of the coral skeletal organic matrix (SOM), they enabled the identification of several skeletal matrix proteins, making *A. millepora* into an emerging model for biomineralization studies. Here we describe the skeletal microstructure of *A. millepora* skeleton, together with a functional and biochemical characterization of its occluded SOM that focuses on the protein and saccharidic moieties. The skeletal matrix proteins show a large range of isoelectric points, compositional patterns and signatures. Besides secreted proteins, there are a significant number of proteins with membrane attachment sites such as transmembrane domains and GPI anchors as well as proteins with integrin binding sites. These features show that the skeletal proteins must have strong adhesion properties in order to function in the calcifying space. Moreover this data suggest a molecular connection between the calcifying epithelium and the skeletal tissue during biocalcification. In terms of sugar moieties, the enrichment of the SOM in arabinose is striking, and the monosaccharide composition exhibits the same signature as that of mucus of acroporid corals. Finally, we observe that the interaction of the acetic acid soluble SOM on the morphology of *in vitro* grown CaCO_3_ crystals is very pronounced when compared with the calcifying matrices of some mollusks. In light of these results, we wish to commend *Acropora millepora* as a model for biocalcification studies in scleractinians, from molecular and structural viewpoints.

## Introduction

In scleractinian corals, the formation of skeletal tissues (*i.e.* skeletogenesis), occurs through a process of biomineralization where the final product – the skeleton – is mainly composed of calcium carbonate in the form of aragonite and of a minor occluded organic fraction (0.07–0.3 wt% [1]–[3]), the skeletal organic matrix (SOM). Although the complete set of mechanisms and pathways involved in coral calcification is yet to be clarified [4]–[8], it is well-established that skeletogenesis in scleractinian occurs externally to the living tissues but is regulated at cellular and molecular levels: at the cellular level, by specialized mineralizing cells, the calicoblastic cells that form a thin ectodermic epithelium covering the skeleton; at the molecular level, by the secretion by this epithelium of the SOM together with the inorganic ionic precursors of mineralization (calcium, bicarbonate) [9]–[15], in the extracellular space comprised between the calicoblastic epithelium and the growing skeleton, the subcalicoblastic space [8], [16].

The SOM - a complex amalgamate of proteins, glycoproteins and polysaccharides - is thought to play a major role in the biomineralization process at different levels, by favoring mineral nucleation and growth, by stopping crystal growth and by spatially organizing the elementary crystallites in well-defined microstructures [17], [18].

Contrarily to mollusks, the degree of control exerted by corals to produce their skeleton has long been debated. At the macroscopic level, corals indeed exhibit a certain plasticity of their skeletal tissues, in relation to environmental parameters [19], while, at the microstructural level, the aragonite biominerals secreted by corals resemble more that produced abiotically [20]. It has been suggested that the mechanism of formation of skeletons in corals is a physiochemically dominated process [21]. This view is however challenged by the complex hierarchical organization of skeletal crystallites observed in all scleractinians [22]–[24]. At a first level, the skeleton is mainly composed of vertical (epitheca, septotheca, septa) and transverse elements (basal plate, dissepiments) [25] built from the skeleton microstructural units - aragonitic fibers that exude from centers of calcification (COC) [26], [27] in a clinogonal orientation (sclerodermites) [21], [28]–[31]. Both fibers and COC were shown to vary in position and proportion among several coral species [22]. Moreover these structural units differ chemically and biochemically [22], [29], [30], [32], [33], a finding that pleads in favor of a strong biological control during skeletogenesis. In view of these results, new biomineralization models were proposed [8], [23], [33], [34], which diverge from earlier models of skeleton growth [21], [26], [35].

Another point in case is the characterization of the skeletal matrix: old biochemical analyses of the bulk, *i.e.* non-fractioned, SOM have shown that the amino acid composition varies from species to species with the most abundant amino acids being aspartic acid, followed by glutamic acid and glycine [1], [36]–[41]. This trend was confirmed by more recent analysis [2], [3], [15], [42], [43] that are in agreement with classical models that attribute to Asp and Glu-rich proteins key-functions in calcium carbonate biomineralization because of their ability to bind reversibly free calcium ions and to interact with a high affinity to calcium carbonate crystal surfaces [17], [44].

Another important characteristic of the SOM is the presence of saccharidic moieties, associated or not to the protein fraction [15], [36], [39], [45]–[47]. The few analyses of the monosaccharides in coral skeletons performed so far suggest that sugar contents can vary considerably and that the monosaccharide composition might be taxon-specific [39]–[41]. Generally speaking, saccharidic fractions are considered, together with the acidic proteins, as key elements in the control of biomineral growth [48], although this remains to be demonstrated for corals. In addition, the presence of lipids has also been reported [42], [49]–[51].

Until recently only two coral skeletal proteins had their primary structure fully characterized – galaxin [52], from the scleractinian *Galaxea fascicularis*, and scleritin [53], from the octocoral *Corallium rubrum*. These proteins were isolated by electrophoresis or chromatography, and partially sequenced through mass spectrometry or Edman degradation. Subsequently the partial protein sequences were either used to build degenerate primers for amplification of protein-encoding transcripts or to parse EST libraries, in order to obtain the full-length sequences. These one-per-one protein approaches are nowadays being challenged by high-throughput techniques, such MS/MS based protein identification in combination with the interrogation of transcriptome or genome data, allowing the identification of a broad set of biomineral-associated proteins. These ‘omic’ approaches reveal not only expected proteins (acidic, galaxins) but also a large repertoire of novel sequences [3], [43]. This recent wealth of information should be the starting point for generating more accurate molecular models of coral skeletal biomineralization.

In the present paper that follows our analysis of skeletal proteins sequences [3], we make a structural, compositional and functional characterization of the skeleton from the staghorn hermatypic coral *Acropora millepora* together with a molecular characterization of the SOM. *Acropora millepora* is a model for coral development [54] with substantial sequence data (genes and transcripts) available. In particular this species has been used to study the stages of settlement and metamorphosis upon the initiation of calcification [55], [56] and is now an emerging model for biomineralization studies [3].

## Materials and Methods

### Sample collection and cleaning

Adult colonies of *Acropora millepora* were collected at the coast of Orpheus Island, Queensland, Australia (Great Barrier Reef Marine Park Authority Permit No G10/33174.1), prior to spawning in November 2010 and transported to the aquaculture facilities at James Cook University in Townsville, where they were maintained in outdoor flow-through aquaria. Mother colonies that died after spawning were used to collect the skeletal material. Animal tissue, symbionts and other microorganisms were removed by immersion in NaOCl (5%, vol/vol) for 72 h. The skeletons were imported to the Netherlands under CITES registration number NL001 (Netherlands Centre for Biodiversity Naturalis).

The skeletal material was then rinsed with milli-Q water, dried and mechanically fragmented, before being reduced to powder in a grinding mill (Fritsch Pulverisette 14). The resulting powder was subsequently sieved (200 µm cutoff). The powder was resuspended and bleached again in NaOCl diluted 10 times (0.26% active chlorine) for 5 h and washed several times with Milli-Q water until no trace of NaOCl was left. The clean powder was dried at 37°C. This treatment allows removing organic exogenous or endogenous contaminants [57], while keeping intact the most tightly bound skeletal matrix components [58].

### Microstructural analysis

The skeleton was observed with a Hitachi TM-1000 tabletop scanning electron microscope (SEM) under a fixed acceleration voltage of 15 kV, in back-scattered electron mode, without carbon sputtering. More detailed microstructural observations were performed with a Field Emission Scanning Electron Microscope (FE-SEM, JSM-7600 F) under an acceleration voltage of 15 kV. To this end, transverse and longitudinal sections were prepared, mirror polished (alumina 0.05 µm, Buehler micropolish), cleaned by repeated sonication in distilled water and dried. In addition, some of the preparations were slightly etched with EDTA (1% w/vol, 3 min.), rinsed with distilled water and dried. Samples were coated with carbon (16 nm), or alternately with gold (15–20 nm). To ensure that the skeletal parts were homogeneous from a crystallographic viewpoint, X-ray energy dispersive spectrometry (EDS) was performed using an OXFORD INSTRUMENTS “INCA ENERGY 350” with XMAX 80 mm2 detector (connected to a Field Emission Scanning Electron Microscope (FE-SEM) JEOL JSM 7600F operating at 15 kV).

### Skeletal organic matrix extraction

The extraction was performed according to a published procedure [59] at 4°C. In brief, the dried powder put in suspension in cold water was decalcified overnight by progressively adding cold dilute acetic acid (10% v/v, 100 µL every 5 sec.) with an electronic burette (Titronic Universal, Schott, Mainz, Germany). The solution was then centrifuged: the organic pellet (acid-insoluble matrix, AIM) was rinsed several times with Milli-Q water and freeze-dried. The volume of the supernatant (acid-soluble matrix, ASM) was reduced by ultrafiltration (Amicon cell 400 mL, 10 kDa cutoff membrane) to about 10 mL, and this solution was dialyzed (Spectra/Por dialysis tube) three days against 1 L water (6 changes) at 4°C and freeze-dried. The extraction was performed in duplicate (2×30 g of skeletal powder under the same NaOCl treatment) in order to check the reproducibility of the results.

### Organic matrix characterization on mono-dimensional gels and Ca-overlay test

The skeletal matrix – both ASM and AIM fractions - was analyzed by conventional mono-dimensional SDS-PAGE (Bio-Rad, miniProtean III). The ASM was directly denatured with Laemmli buffer [60] according to standard conditions while the AIM was only partly solubilized by the Laemmli buffer, giving a LS-AIM fraction (Laemmli-Soluble, Acid-Insoluble Matrix). Gels were stained with silver nitrate [61]. Calcium-binding capacity of both fractions was investigated via the ^45^Ca overlay test [62]: the 1D-gel was transferred on a PVDF Immobilon-P membrane (Millipore Corp.) with a mini-Trans Blot module (Bio-Rad) for 90 min at 120 mA. Subsequently the membrane was incubated 3 times 20 min in an overlay buffer (60 mM KCl, 5 mM MgCl_2_, 10 mM imidazole-HCl, pH 6.8), then 30 min in the same buffer containing ^45^CaCl_2_ (40 MBq/liter, 1.4 µM). After a brief washing (50% ethanol, 2×2 min), the membrane was air-dried and exposed to a film (X-Omat, Kodak) for 1 week. The calcium-binding protein calmodulin (CaM) (catalog number P2277; Sigma) was used as a positive control.

### Analysis of the protein content of the SOM

LC-MS/MS data for the AIM and ASM were acquired and analyzed as described in [3]. Further characterization of the mature protein sequences was performed with the tool ProtParam (http://web.expasy.org/protparam/) to predict theoretical molecular weights, AA composition and isoelectric point. Putative post-translational modifications (PTMs) and other functional sites were identified with the ELM server (http://elm.eu.org/), peptide signals with SignalP (http://www.cbs.dtu.dk/services/SignalP/), transmembrane helices with TMHMM (http://www.cbs.dtu.dk/services/TMHMM/) and GPI () anchor sites with (http://mendel.imp.ac.at/gpi/cgi-bin/gpi_pred.cgi).

### Analysis by Fourier Transform Infrared Spectrometry (FTIR-ATR) and by Raman spectroscopy

FTIR spectra were recorded on a Fourier Transform IR spectrometer Bruker Vector 22 (Karlsruhe, Germany) equipped with a Golden Gate Attenuated Total Reflectance (ATR) device (Specac Ltd., Orpington, UK) in the 4000–500 cm^−1^ wavenumber range (ten scans with a spectral resolution of 2 cm^−1^). Analyses were carried out on the cleaned skeletal powder, on the lyophilized samples from AIM and ASM (<1 mg) and on the dried samples from *in vitro* crystallization tests (see below). In this latter case, crystals were gently detached from the glass plate with s sterile scalpel and directly transferred on the quartz window of the spectrophotometer. Assignment of absorption bands was performed by comparison with descriptions of previous spectra available in the literature [70].

In addition to FTIR characterization, Raman spectroscopy was used, in particular for checking the mineralogy of the skeletal powder and of the crystals produced *in vitro* (see below). Samples were placed under the binocular of a Renishaw inVia Raman microscope and carefully centered. Spectra were then recorded using a 633 nm He-Ne laser. The samples were scanned for 1000 ms in a scan range of 1015 cm^−1^. The spectroscopic profiles of samples were compared to that of standards of biogenic calcite, aragonite and vaterite.

### Sugar analysis

The presence of saccharidic moieties was checked qualitatively and quantitatively by two complementary techniques: FTIR (see above), and monosaccharide analysis by high performance anion exchange with pulsed amperometric detection (HPAE-PAD). For quantifying the monosaccharide content, lyophilized samples of ASM and AIM were hydrolyzed in trifluoroacetic acid (TFA) (2 M) at 105°C for 4 h. Evaporation for complete dryness of the samples (1 h) was performed, before dissolving and homogenizing in NaOH (100 µL, 20 mM). The samples were then centrifuged and the supernatant (10 µL) injected on a CarboPac PA100 column (Dionex Corp., Sunnyvale, CA, USA) at 5, 25 and 50 mg/L. Carbohydrate standard (Sigma, St Louis, MO, USA) was injected at 16, 8, and 4 ppm. The same method was applied to non-hydrolyzed samples in order to detect free monosaccharides that could have contaminated the sample during dialysis.

Note that the hydrolytic conditions do not allow the quantification of GalNAc and GlcNac, which are converted into GalN (galactosamine) and GlcN (glucosamine), respectively. Moreover sialic acids are destroyed during the hydrolytic procedure and were not quantified here.

### *In vitro* crystallization tests in the presence of ASM

The interaction of the ASM with CaCO_3_ was tested *in vitro*. Several dilutions of a concentrated mother solution of ASM (4 µg/µL) were prepared on a 10 mM CaCl_2_ solution (pH 7) in a range of concentrations from 0.1 to 20 µg/mL. A volume of 0.2 mL from each dilution was introduced in sterile sixteen-well chamber slide system (Lab-Tek, Nunc, Rochester, NY, USA), the plastic cover of which was formerly pierced for each well (1 mm holes). The chamber slide system was consequently sealed with parafilm and placed at 4°C. in a closed desiccator containing crystals of ammonium bicarbonate. Negative controls were made with a 10 mM CaCl_2_ solution without ASM. After 46 h incubation, the solutions were removed by suction and the wells were dried and observed at 15 keV on a Hitachi TM-1000 Table-top Scanning Electron Microscope (SEM). The nature of the crystals was checked by FTIR and Raman spectroscopy (see above).

## Results

### Skeletal morphology and microstructure

**Figure 1:**
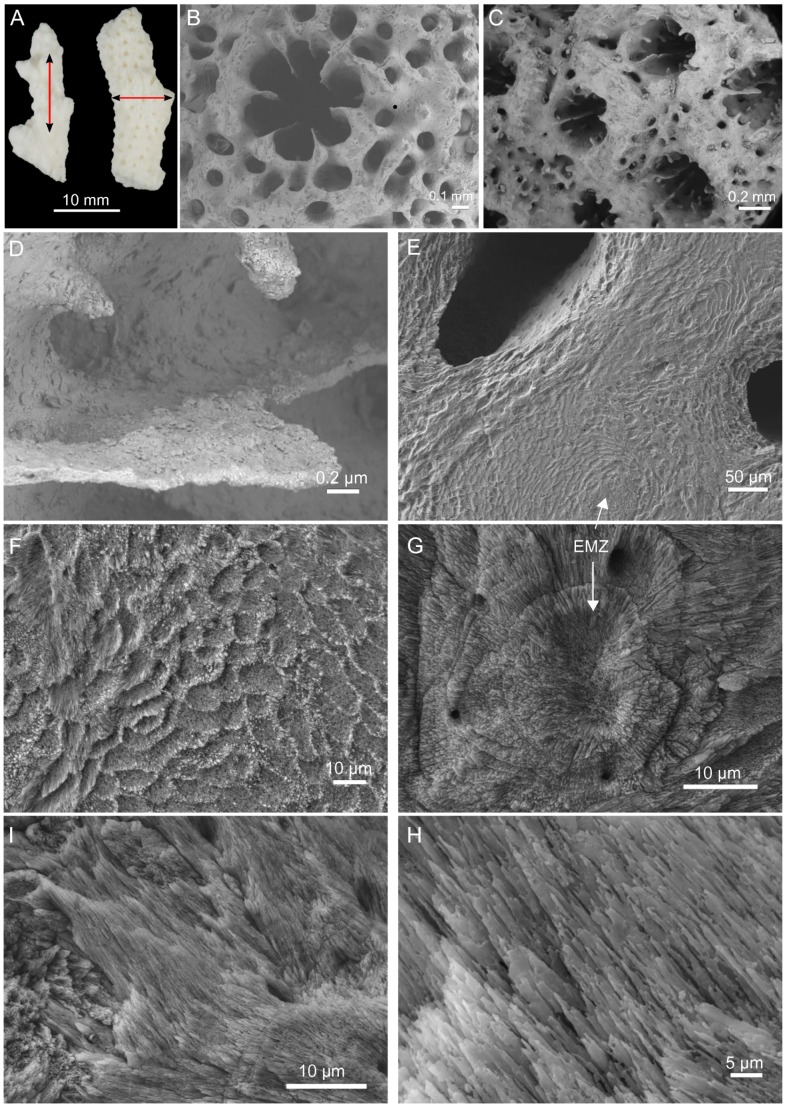
Skeleton morphology and microstructure. (A) Skeletal fragments after treatment in NaOCl (5%, vol/vol) for 72 h prior to longitudinal and transversal cuts. Scanning electron microscopy images from the skeleton morphology: (B) Axial corallite, (C) Radial corallites, (D) Closer view into a radial corallite showing different septa. Polished and EDTA-etched sections from a transversal cut (E–G) and longitudinal cut (H–I). EMZ – early mineralization zone.

Prior to further characterizations, the aragonitic nature of the skeleton of *Acropora millepora* was confirmed by Raman and FTIR spectra (Figure S1) in order to ensure the absence of recrystallization in calcite, or the presence of traces of vaterite. In addition, EDS spot analyses were performed on different points (transects, and points taken randomly, data not shown) for checking the elemental homogeneity of the skeleton. They confirmed that the atomic percentages of minor elements (Mg, Na, Sr, S) did not vary significantly, demonstrating that the skeletal parts were fresh from top to bottom and also in their diameter (see Figure 1 A). This check allowed us to assert with confidence that the fragments used for the subsequent matrix extraction were not submitted to detectable early diagenetic processes, affecting the mineral phase.

From a morphological viewpoint, the *A. millepora* skeleton has cup-like corallites with separate walls and a highly porous coenosteum. At the top, the axial corallite shows six complete radiating septa (Figure 1 B). The corallites are evenly distributed and sized along the skeletal surface (Figure 1 C) with complete and incomplete septa (Figure 1 D). A first view of a polished and etched transverse section (Figure 1 E) reveals a shingle-like microstructure with unevenly distributed porosity. A closer look at the same surface highlights a layer of “centers of calcification” that have been later termed as early mineralization zones, EMZ [23] (Figure 1 F) and from which fibers diverge in trabecular orientation (Figure 1 G) [27]. These two skeletal components, i.e. EMZ and fibers, account for the basic units of the coral skeletal microstructure.

Closer views of polished and etched longitudinal sections show the crystalline needle-like fibers (Figure 1 H and I). In Figure 1 H, it is possible to observe different clusters of fibers in distinct layers and directions. Each cluster diverges from its EMZ, which in turn nucleates at random positions in the growing skeleton causing the multiple directions of clusters of fibers observed.

### Skeletal organic matrix on gel

**Figure 2:**
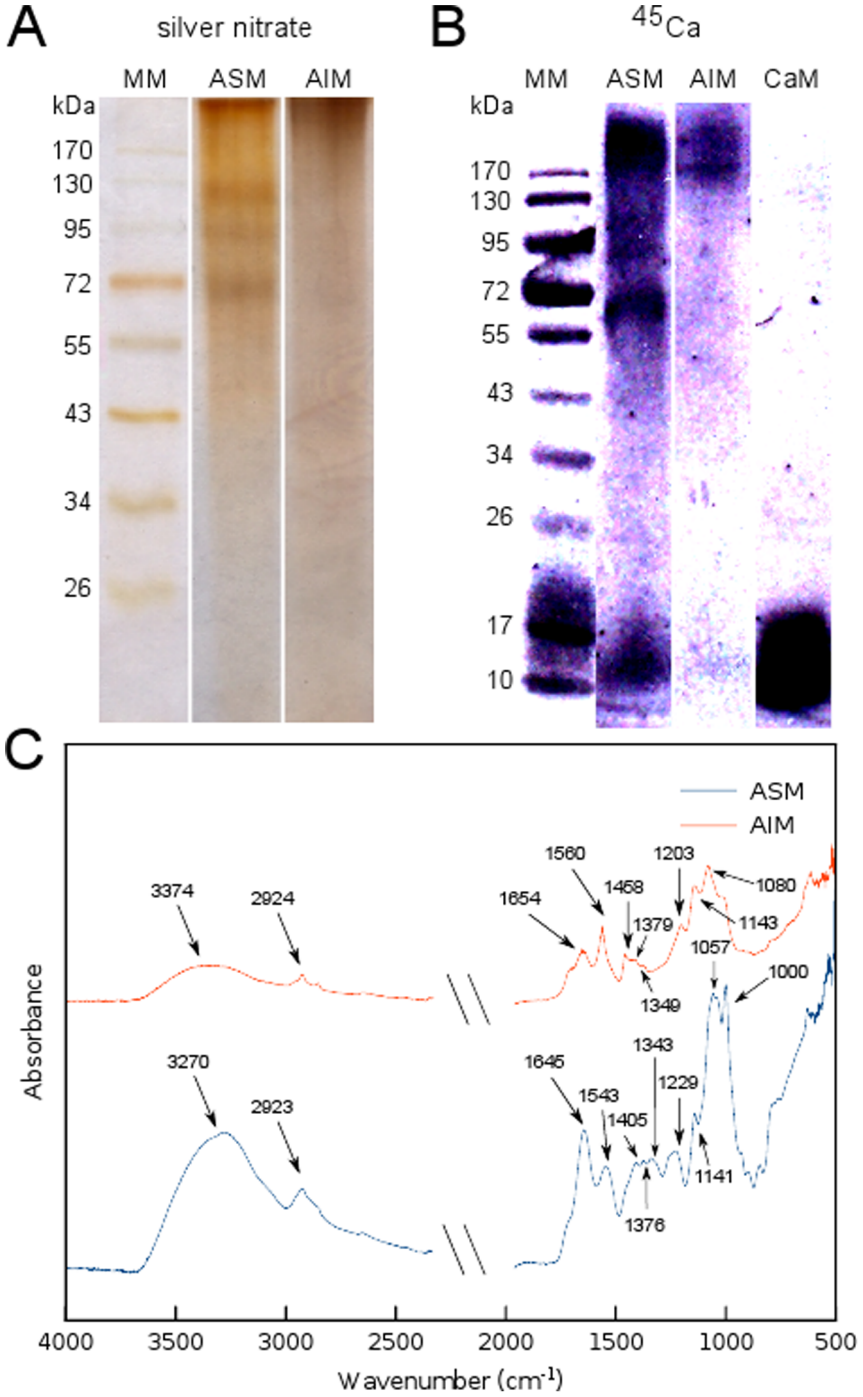
Molecular composition of the skeletal organic matrix from A. millepora. (A) Analysis of electrophoresis on gel after AgNO3 staining.(B) PVDF membrane revealed by autoradiography with 45Ca, calmodulin (CaM)was used as positive control. (C) Infrared absorption spectra of ASM and AIM fractions with assignment of the main peaks. MM – Molecular marker, ASM – Acid soluble matrix, AIM – Acid insoluble matrix.

The extraction of the skeletal organic matrix from fine skeleton powder yielded 0.034±0.01% of ASM and 0.23±0.03% of AIM (w/w of the dry powder), corresponding to a AIM/ASM ratio between 6 and 7. The analysis of the ASM on mono-dimensional gel (Figure 2 A) stained with AgNO_3_ revealed a profile comprising 3 main diffuse bands at approximately 120, 90 and 64 kDa in a smear of polydisperse macromolecules that stains preferentially around high molecular weights. Between 20 and 40 kDa, the ASM is hardly stained or tends to stain negatively. On AgNO_3_-stained gel, the profile of the AIM is uniformly stained and shows exclusively polydisperse molecules. Both fractions exhibit a calcium-binding ability, when labeled with ^45^Ca (Figure 2 B). In the ASM, the 64 kDa band shows a strong Ca-binding signal while two other non discrete signals are detected for high (above 170 kDa) and low (below 17 kDa) molecular weights components. In addition, the two other discrete bands (at 120 and 90 kDa respectively) exhibit fainter Ca-binding signals. In the AIM, only a significant signal is observed at high molecular weights (above ∼172 kDa). This signal is concentrated in a short smear and in two distinct discrete bands, which in turn are not stained with silver nitrate.

### Fourier transform IR of the ASM and AIM

FTIR (ATR) spectra were acquired for both skeletal organic matrix fractions, ASM and AIM (Figure 2 C). Characteristic absorption bands corresponding to the protein backbone bonds [63], [64] are observed at 3270 and 3374 cm^−1^ (amide A, νN-H), at 1645 and 1654 cm^−1^ (amide I, νC = O), at 1543 and 1560 cm^−1^ (amide II, νC-N) and also at 1376–1343 and 1379–1203 cm^−1^ (amide III, several modes in the region of 1400–1200 cm^−1^), for the ASM and AIM respectively. The weaker absorptions at 2923 (ASM) and 2924 cm^−1^ (AIM) are consistent with νC-H stretching vibrations [65]. In the ASM spectrum a distinct band appears at 1229 cm^−1^, which usually relates to the presence of sulfate groups (S = O), suggesting that the polysaccharides are sulfated [48]. The bands located in the range of 1140–1000 cm^−1^ can be indicative of saccharidic moieties and attributed to C-C-O and C-O-C stretching elongations [65]. Note that these absorptions are extremely intense in the both spectra, but in particular for the ASM, being considerably higher than the peaks corresponding to the peptide bonds.

### Monosaccharide composition of the ASM and AIM

**Figure 3:**
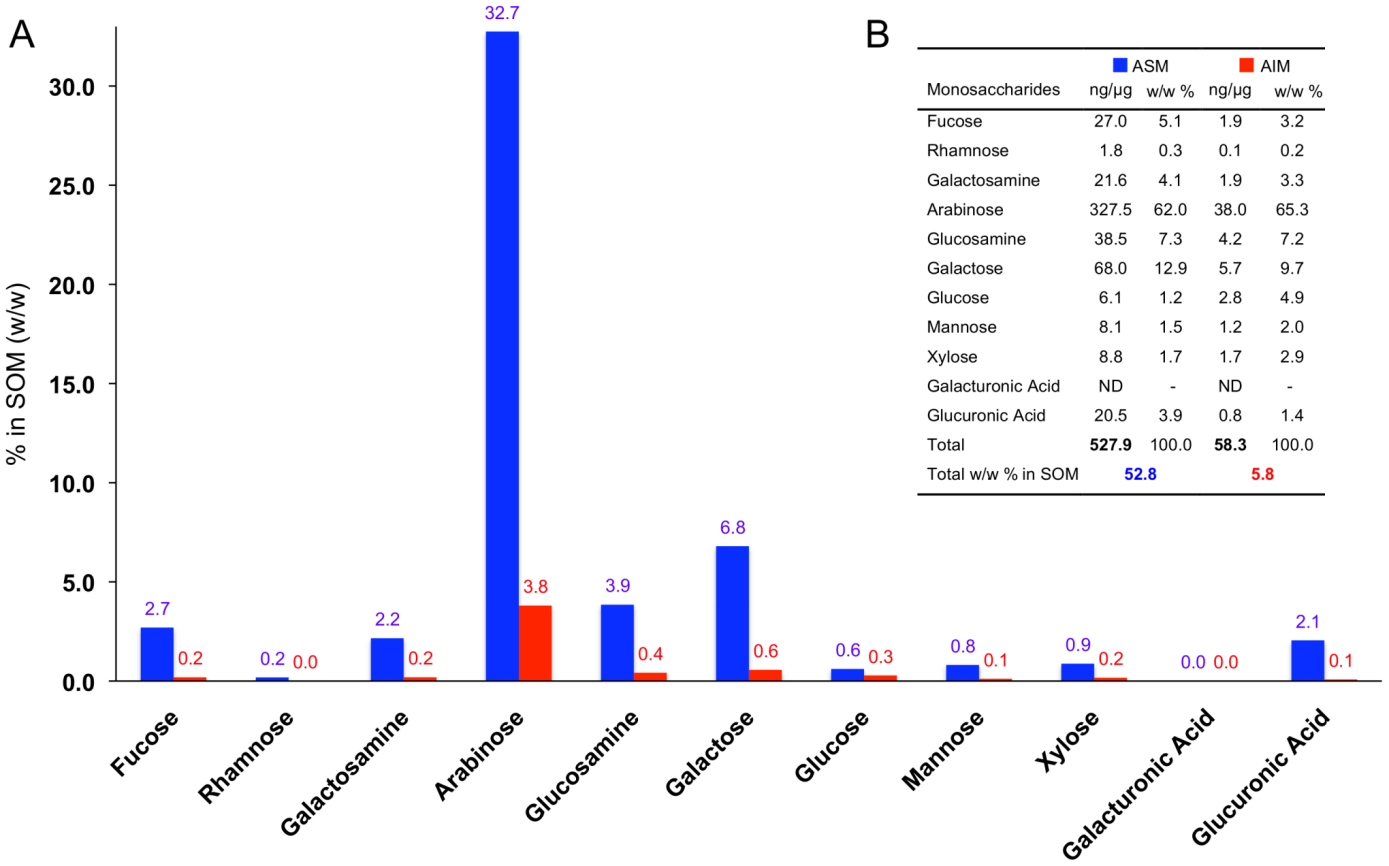
Quantification of neutral, aminated and acidic monosaccharides in the ASM (blue) and in the AIM (red) of *A. millepora*. Samples were hydrolyzed with 2°C (4 h). (A) Total Wt. % in the skeletal organic matrix (SOM, either ASM or AIM) are indicated in the graph (bars). (B) Concentrations (ng/µg) and relative molar percentages are shown in the table for both matrices.

The quantification of the monosaccharide fraction in the ASM and AIM confirms the results of the FTIR spectra, i.e. that both matrices are glycosylated (Figure 3) and comprise neutral, amino and acidic monosaccharides.

In the ASM, the absolute quantification of the monosaccharide content indicates that the glycosyl moieties constitute more than half of this fraction, 52.8% (w/w). The proportion of monosaccharides in the AIM is, by contrast, considerably less and represents only 5.8% (w/w) of this fraction. However, for this fraction, we cannot exclude that this percentage is underestimated. Indeed, the extraction procedure by mild standard hydrolysis (TFA 2 M, 105°C, 4 hrs) left an insoluble residue at the end of the procedure that might still contain high amount of carbohydrates.

In both matrices, we observed that the compositional profiles (w/w %) could be almost superimposed for each monosaccharide (table in Figure 3). In particular, the most abundant monosaccharide was by far arabinose, constituting 62% and 65.3% w/w of the total sugar moiety, in the ASM and AIM respectively.

Far behind, galactose represents the second most abundant monosaccharide (12.9% in the ASM and 9.7% in the AIM), followed by glucosamine (7.3% in the ASM and 7.2% in the AIM), fucose (5.1% in the ASM and 3.2% in the AIM) and galactosamine (4.1% in the ASM and 3.3% in the AIM). The amount of these sugars in the total weight of SOM was nine times higher (527.9 ng/µg versus 58.3 ng/µg) in the ASM than in the AIM (Figure 3, histogram and Table). Rhamnose, glucose, mannose and xylose represent extremely minor sugars in both fractions. The proportion of glucose is higher in the AIM (4.9%, against 1.2% in the ASM). On the contrary, the ASM exhibits an appreciable proportion of glucuronic acid (3.9%), while this sugar is depleted in the AIM. Galacturonic acid is detected in none of the two extracts.

### Characterization of skeletal organic matrix proteins (SOMPs)

By combining MS-based proteomic data obtained for the ASM and AIM with the transcriptomic data publicly available for *A. millepora*, a total of 36 SOMPs were identified [3] (Table 1). These proteins enter in few groups, which have been described previously, according to their primary structure [3]: acidic proteins, proteins with an extracellular matrix signature, enzymes, proteins with transmembrane domains, ‘orphan’ proteins, i.e., proteins that cannot be simply affiliated to a specific group or function, and finally, one toxin.

**Table 1:** Characterization of the 36 skeletal organic matrix proteins (SOMPs).

UniprotKB accession	Name (Abbrev.)	Number of residues	Mw* (KDa)	pI*	Major*	Putative PTM	Other sequence properties
number		(status)			aa (%)		
B3EWY6	Skeletal acidic Asp-rich Protein 1 (SAARP 1)	386	40.1	3.92	Asp (20.4)	Glycosaminoglycan site (2), N-glycosylation (3), Phosphorylation: Ser (22), Thr (5), Tyr (3)	SP [1]–[24], GPI anchor (S, 359), Asp-rich [60–108, 260–288], ND repeat [67–103]
B3EWY7	Acidic skeletal organic matrix protein (Acidic SOMP)	359	36.1	4.13	Asp (9.9)	Glycosaminoglycan site (6), N-glycosylation (5), O-Fucosylation (1), Phosphorylation: Ser (18), Thr (5), Tyr (2)	SP [1]–[26], GPI anchor (N, 333), Asp-rich [61–86, 246–262, 341–355]
B3EWY8	Skeletal acidic Asp-rich Protein 2 (SAARP2)	390 (Fragment)	42.4	4.24	Asp (21.1)	Glycosaminoglycan site (2), N-glycosylation (3), Phosphorylation: Ser (15), Thr (6), Tyr (1)	SP [1]–[16], TM [367–389], Asp-rich [48–83, 91–124, 271–292], DDK repeat [97–107]
B3EWY9	Mucin-like	1594 (Fragment)	-	-	-	ASX hydroxylation (3), Glycosaminoglycan site (7), N-glycosylation (12), O-Fucosylation (1), Phosphorylation: Ser (47), Thr (25), Tyr (9)	TM [1531–1553]
B3EWZ0	Secreted acidic protein 1 (Amil-SAP1)	168 (Fragment NT)	-	-	-	Glycosaminoglycan site (4) N-glycosylation (1) Phosphorylation: Ser (7), Thr (4), Tyr (0)	SP [1]–[20], GPI anchor (G, 119), RGD motif [119–121]
B3EWZ1		142 (Fragment CT)	-	-	-	Glycosaminoglycan site (2) N-glycosylation (1) Phosphorylation: Ser (4), Thr (0), Tyr (2)	TM [124–141], G[D,7]S repeat [139–168]
B3EWZ2	Uncharacterized skeletal organic matrix protein-8 (USOMP-8)	214	20.9	5.26	Ser (8.9)	Glycosaminoglycan site (2) N-glycosylation (3)	SP [1]–[24], LCR [119–133]
B3EWZ3	Coadhesin	1675 (Fragment)	-	-	-	Peptide C-terminal amidation (1) Glycosaminoglycan site (5) Phosphorylation: Ser (60), Thr (30), Tyr (13)	TM [1361–1383]
B3EWZ4	Secreted acidic protein 2 (Amil-SAP2)	168 (Fragment)	-	-	-	Glycosaminoglycan site (6), Phosphorylation: Ser (11), Thr (2), Tyr (3)	SG[D,6]GD repeat [4]–[33]
B3EWZ5	MAM and LDL-receptor domain- containing protein 1	5145 (Fragment)	-	-	-	Glycosaminoglycan site (3), N-glycosylation (1), Phosphorylation:?	RGD motif [47]–[49], P[T,2] repeat [1099–1110], [P,2][T,2] repeat [2512–2522]
B3EWZ6	MAM and LDL-receptor domain- containing protein 2	7311 (Fragment)	-	-	-	Glycosaminoglycan site (6), N-glycosylation (2), O-Fucosylation site (1), Phosphorylation:?	P[T,2] repeat [178–189], [P,2][T,2] repeat [2261–2271]
B3EWZ7	Threonine-rich protein	288 (Fragment)	-	-	-	Glycosaminoglycan site (2), N-glycosylation (10), Phosphorylation: Ser (9), Thr (30), Tyr (0)	SP [1]–[21], Thr-rich [151–262], TEAP[T,2] repeat [168–261]
B3EWZ8	Ectin	400 (Fragment)	-	-	-	Glycosaminoglycan site (4), N-glycosylation (1), Phosphorylation: Ser (17), Thr (7), Tyr (5)	SP [1]–[21]
B3EWZ9	Hephaestin-like	1114	122.0	5.83	Gly (8.5)	Peptide C-terminal amidation (3), Glycosaminoglycan site (5), N-glycosylation (2), Phosphorylation: Ser (28), Thr (17), Tyr (14)	SP [1]–[26], TM [1090–1112], GPI anchor (A, 1090)
B3EX00	Uncharacterized skeletal organic matrix protein-1 (USOMP-1)	448 (Fragment)	-	-	-	Glycosaminoglycan site (5), N-glycosylation (8), Phosphorylation: Ser (19), Thr (6), Tyr (0)	LCR [101–120, 318–344, 428–448]
B3EX01	CUB domain-containing protein	409	42.8	5.05	Thr (13.6)	Glycosaminoglycan site (3), N-glycosylation (6), Phosphorylation: Ser (17), Thr (17), Tyr (5)	SP [1]–[18], LCR [91–104, 150–229, 349–359, 392–409]
B3EX02	MAM and fibronectin- containing protein	422 (Fragment)	-	-	-	Peptide C-terminal amidation (1), Glycosaminoglycan site (3), N-glycosylation (6), Phosphorylation: Ser (18), Thr (4), Tyr (5)	
B7W112	Glu-rich protein	522	58.3	3.96	Glu (22.3)	Glycosaminoglycan site (1), Phosphorylation: Ser (35), Thr (5), Tyr (10)	SP [1]–[16], Glu-rich [107–134, 152–201, 227–262], DEAE repeat [358–425]
B7W114	Cephalotoxin-like protein	473 (Fragment)	-	-	-	Glycosaminoglycan site (5), N-glycosylation (1), Phosphorylation: Ser (16), Thr (3), Tyr (8)	SP [1]–[21]
B7WFQ1	Uncharacterized skeletal organic matrix protein-2 (USOMP-2)	505	52.9	5.90	Cys (10.5)	Peptide C-terminal amidation (1), Glycosaminoglycan site (7), N-glycosylation (7), Phosphorylation: Ser (12), Thr (6), Tyr (3)	SP [1]–[19]
B8RJM0	Uncharacterized skeletal organic matrix protein-3 (USOMP-3)	433 (Fragment)	-	-	-	Glycosaminoglycan site (6), N-glycosylation (2), Phosphorylation: Ser (22), Thr (8), Tyr (5)	SP [1]–[28], TM [275–297]
B8UU51	Galaxin 2	275	26.8	8.18	Cys (11.8)	Glycosaminoglycan site (2), N-glycosylation (2), Phosphorylation: Ser (4), Thr (2), Tyr (3)	SP [1]–[20], 5 di-Cys repeats
B8UU59	polycystic kidney disease 1-related skeletal organic matrix protein (PKD1-related protein)	3029 (Fragment)	-	-	-	Peptide C-terminal amidation (2), Glycosaminoglycan site (45), N-glycosylation (46), O-Fucosylation site (2), Phosphorylation: Ser (130), Thr (37), Tyr (31)	SP [1]–[21], TM [1684–1706, 1896–1913, 1933–1955, 2103–2125, 2140–2162, 2250–2272, 2457–2479, 2491–2513, 2545–2564, 2585–2607, 2651–2673], RGD motif [2852–2854]
G8HTB6	Zona pellucida domain-containing protein	414	43.8	4.92	Ser (10.3)	Glycosaminoglycan site (3), Phosphorylation: Ser (18), Thr (7), Tyr (7)	SP [1]–[17], TM [366–388], LCR [39]–[64]
B8UU74	Uncharacterized skeletal organic matrix protein-4 (USOMP-4)	204 (Fragment)	-	-	-	Phosphorylation: Ser (3), Thr (3), Tyr (1)	LCR [17]–[34]
D9IQ16	Galaxin	338	32.7	5.15	Cys (12.7)	Glycosaminoglycan site (1), N-glycosylation (1), Phosphorylation: Ser (7), Thr (1), Tyr (3)	SP [1]–[23], 9 di-Cys repeats
B8UU78	EGF and laminin G domain-containing protein	1124	123.8	6.38	Gly (8.5) Ser (8.5)	ASX hydroxylation (1), Glycosaminoglycan site (16), N-glycosylation (3), Phosphorylation: Ser (42), Thr (14), Tyr (17)	TM [1056–1078], LCR [423–434, 1110–1121]
B8V7P3	Putative carbonic anhydrase	148 (Fragment)	-	-	-	Glycosaminoglycan site (1), Phosphorylation: Ser (2), Thr (2), Tyr (0)	
B8V7Q1	Protocadherin-like	4467	486.1	4.98	Val (9.7)	Peptide C-terminal amidation (1), Glycosaminoglycan site (27), Phosphorylation?	SP [1]–[22], TM [4257–4279], NGR motif [1515–1517, 3798–3800] [S,2][G,2]SVGV[S[G,2],2]ASV[G,2]SI[G,2], ASG repeat [4092–4136], ILV[I,2]GA repeat [4263–4276]
B8V7R6	Collagen alpha-1 chain	888 (Fragment)	-	-	-	Peptide C-terminal amidation (3), Glycosaminoglycan sites (17), N-glycosylation (3), Phosphorylation: Ser (36), Thr (9), Tyr (7)	LCR [98–114, 225–279, 302–331, 422–441, 459–489], G[P,2] repeat [609–621], NGR motif [413–415, 452–454], RGD [221–223]
B8V7S0	CUB and peptidase domain-containing protein 1	435 (Fragment)	-	-	-	N-glycosylation (2), Phosphorylation: Ser (11), Thr (11), Tyr (6)	LCR [384–405]
B7T7N1	MAM and fibronectin containing protein 2	112 (Fragment)	-	-	-	Glycosaminoglycan site (4), N-glycosylations (3), Phosphorylation: Ser (3), Thr (1), Tyr (1)	
B8VIV4	CUB and peptidase domain-containing protein 2	389 (Fragment)	-	-	-	Phosphorylation: Ser (4), Thr (3), Tyr (5)	
B8VIU6	Uncharacterized skeletal organic matrix protein-5 (USOMP-5)	256	25.2	8.92	Ser (11.9)	Glycosaminoglycan site (3), N-glycosylations (6), Phosphorylation: Ser (10), Thr (6), Tyr (1)	SP [1]–[21]
B8VIW9	Neuroglian-like	1280	140.4	5.65	Ser (8.0)	Peptide C-terminal amidation (2), Glycosaminoglycan site (2), Phosphorylation: Ser (58), Thr (29), Tyr (17)	SP [1]–[19], TM [4257–4279], NGR motif [1196–1198]
B8VIX3	Uncharacterized skeletal organic matrix protein-6 (USOMP6)	436	48.1	9.04	Glu (14.4)	Peptide C-terminal amidation (1), N-glycosylations (4), Phosphorylation: Ser (19), Thr (9), Tyr (1)	SP [1]–[20], LCR [316–326, 414–427]
B8WI85	Uncharacterized skeletal organic matrix protein-7 (USOMP7)	422	44.3	9.26	Val (9.0)	Glycosaminoglycan site (7), N-glycosylation (1), Phosphorylation: Ser (11), Thr (1), Tyr (10)	SP [1]–[23]

Computed parameters: molecular weight (Mw), isoelectric point (pI), most abundant amino acid (Major aa%), post-translational modifications (PTM) and other sequence features: signal peptide (SP), transmembrane domain (TM), glycosylphosphatidylinositol (GPI anchor), complexity regions (LCR), regions of biased composition, motifs and repeats.

*Properties calculated based on the primary sequence of the mature protein, *i.e.* without peptide signal.

The overall parameters deduced from the mature primary structure of 17 complete proteins indicate that their theoretical pI varies between very acidic (3.92) and basic (9.26). Indeed, 4 proteins exhibit a basic pI (superior to 8), 9 proteins are moderately acidic (pI close or above 5) and 4 are acidic (pI close or below 4). The latter proteins are predominantly enriched in aspartic acid residues; only one Glu-rich protein (B7W112), exhibits high proportion (22.3%) of glutamic acid residues. Interestingly, the uncharacterized skeletal organic matrix protein-6 (USOMP-6, B8V1X3) has also glutamic acid as a major aa component (14.4%), while exhibiting a basic pI. This is mainly due to its high proportion of positively charged residues (Arg+Lys+His = 18.9%), which is superior to that of the acidic ones (Asp+Glu = 17.3%). The theoretical molecular weights vary between 20 and 140 kDa; only in one case, a high molecular weight protein (protocadherin-like  = 486 kDa) was detected.

As indicated in Table 1 (column 7), all these proteins (full-length or fragments) exhibit putative post-translational modifications, in particular N-glycosylations and phosphorylations on Ser, Thr and Tyr residues. In addition most of the proteins exhibit attachment sites for glycosaminoglycans (Ser-Gly-X-Gly-), from 1 site (eg. galaxin, carbonic anhydrase) up to 45 sites (PKD1-related protein), and fewer SOMPs have also putative O-fucosylation sites. However, the reality of these PTMs remains to be demonstrated experimentally.

Other important properties in the 36 sequences include the presence of signal peptides, transmembrane domains, GPI anchors, short motifs – typically three to six amino acid residues - repeated along the sequence, and at last by low complexity regions (LCR) typified by the Asp-rich domains in aspartic acid-rich proteins. Furthermore, functional short motifs have been found which support cell adhesion in many proteins [66], [67], in particular RGD (Arg-Gly-Asp, detected in four proteins) and NGR (Asn-Gly-Arg, detected in three proteins). Both motifs constitute attachment sites for a large number of integrins [68] and consequently support cell-cell and protein-cell interaction in the extracellular matrix.

### *In vitro* interaction of the acetic acid-soluble matrix with CaCO_3_

**Figure 4:**
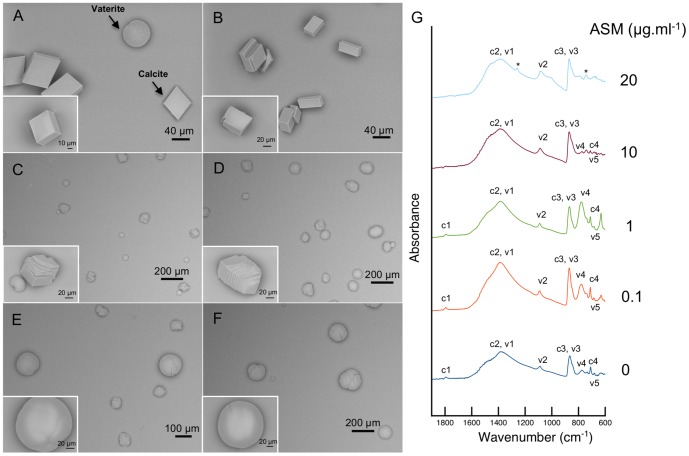
SEM images of CaCO_3_ crystals grown *in vitro* with the addition of different concentrations of ASM: (A) 0 µg.ml^−1^, (B) 0.1 µg.ml^−1^, (C) 1 µg.ml^−1^, (E) 5 µg.ml^−1^, (E) 10 µg.ml^−1^ and (F) 20 µg.ml^−1^. (G) Corresponding FTIR(ATR) absorbance spectra on the whole precipitated for the following ASM concentrations: 0, 0.1, 1, 10 and 20 µg.ml^−1^.

Increasing concentrations of ASM were used in interaction with growing CaCO_3_ crystals in order to measure to which extent the skeletal organic matrix is able to interfere on the morphologies of the produced crystals, in 46-hours incubation experiments (Figure 4). The negative control – crystals grown in the absence of ASM – is shown on Figure 4 A. It reveals the typical rhombohedral calcite, accompanied with few vaterite crystals that exhibit the hexagonal symmetry. The compositional mixture of these two CaCO_3_ polymorphs was confirmed by the FTIR absorption spectra (Figure 4 G), where it was possible to identify the characteristic calcite peaks (c1 – 1793, c3 – 868, c4 – 712 cm^−1^) as well as peaks corresponding to vaterite (v2 – 1092, v3 – 868, v4 – 776, v5 – 685 cm^−1^) [69], [70]. Calcite peaks showed higher intensity since this polymorph was more abundant than vaterite in the control.

The effect of adding low concentrations of ASM was immediately evident in the polymorphs shape. In the same growing conditions as the control, the addition of skeletal organic matrix at 0.1 µg.ml^−1^ produced several crystals with multiple faces instead of regular ones (Figure 4 B) whereas the addition of ASM at 1 µg.ml^−1^ (Figure 4 C) affected all the crystals observed, which developed multilayered faces and rounder edges. Similarly to the control, the FTIR spectra obtained for the crystals grown with 0.1 and 1 µg.ml^−1^ of ASM showed that the polymorph mixture obtained was composed of calcite (peaks c1 – 1793, 1793; c3 – 870, 868; c4 – 712, 712 cm^−1^) and vaterite (peaks v2 – 1092, 1091; v4– 779, 779; v5– 685, 685) (Figure 4 G). At ASM concentrations of 5 µg.ml^−1^ (Figure 4 D) not only crystals with multilayered faces and round edges appeared but also completely round-shaped crystals were observed, i.e. without any defined faces. When increasing the ASM content to 10 µg.ml^−1^, the round crystals were larger and exhibited complete smooth surfaces (Figure 4 E). Some crystals with multilayered faces were still present but in much less number than for 5 µg.ml^−1^. In the infrared spectrum some of the characteristic calcite (c1, c4) and vaterite (v4) absorbance peaks were less intense. At 20 µg.ml^−1^ the samples were characterized by the sole presence of round and smooth crystals (Figure 4 F) and the complete reduction of characteristic calcite (c1, c4) and vaterite (v4) absorbance peaks. Only peaks corresponding to c2 and v1 (1385, 1395 cm^−1^), v2 (1088, 1085 cm^−1^), c3 and v3 (870, 871 cm^−1^) were significant at 10 and 20 µg.ml^−1^. The preservation of these peaks as well as the emergence of two other ones (* - 1259, 742 cm^−1^) may be biased due to the higher concentrations of ASM (Figure 4 G, 20 µg.ml^−1^).

**Figure 5:**
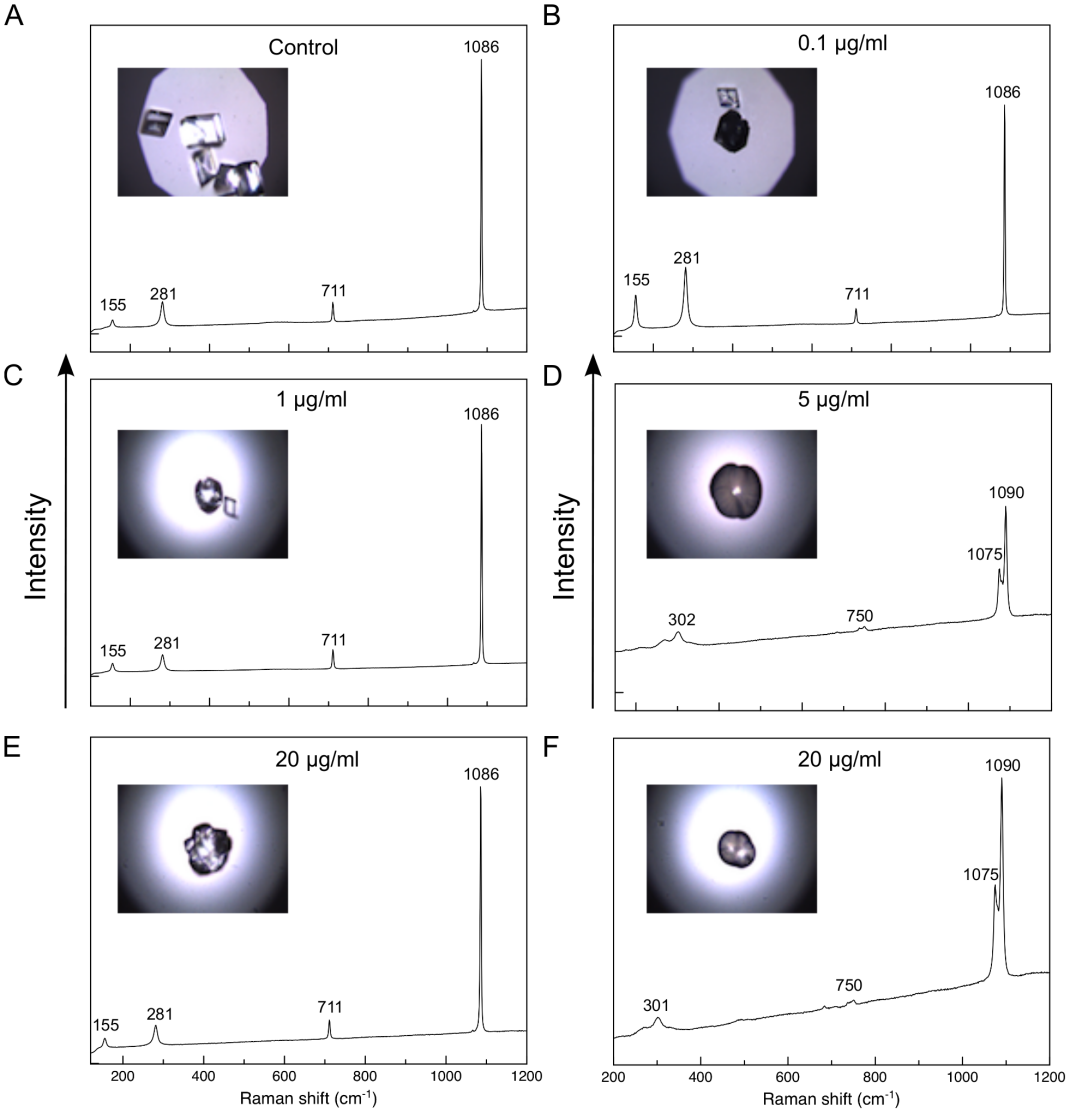
Raman spectra obtained from different crystals grown *in vitro* with the addition of different concentrations of ASM: (A) 0 µg.ml^−1^, (B) 0.1 µg.ml^−1^, (C) 1 µg.ml^−1^, (E) 5 µg.ml^−1^, (E) 20 µg.ml^−1^ and (F) 20 µg.ml^−1^. The visible bands clearly distinguish calcite and vaterite.

In order to double-check which polymorphs are present in the *in vitro* experiments, Raman spectroscopy was used. Spectra were acquired directly on several isolated crystals, and few results were selected for Figure 5. Spectra from crystals of Figure 5 A, B, C, E are typically that of calcite with three peaks at 281, 711 and 1086 cm^−1^, while those shown in Figure 5 D and F are characteristic of vaterite, in particular the peak at 301 cm^−1^ and the double peak at 1075–1090 cm^−1^. Therefore, the polymorphs formed were calcite and vaterite regardless of the concentration of ASM used.

## Discussion

The skeleton of the hermatypic scleractinian coral *Acropora millepora* is fully aragonitic, a finding congruent with former investigations on newly settling recruits of the same species [71]. At the microstructural level the skeleton reveals compacted early mineralization zones randomly disposed that concur with its higher calcification rates in their natural habitat [72]. The *A. millepora* skeleton contains an occluded organic matrix, which is here extracted and biochemically characterized for the first time. This work covers aspects of coral biomineralization that complement our previous study on the sequence analysis and evolution of the *A. millepora* skeletal proteome [3].

Similarly to other matrices associated with calcified skeletons, the matrix of *A. millepora* comprises two fractions, according to their solubility in a decalcifying solution of dilute acetic acid. The acid soluble matrix is a minor fraction that represents about 1/6 of the insoluble one. In total, the SOM accounts for 0.26% of the skeleton weight. This value can be compared with those recently obtained from different scleractinians [2], [73] that vary between 0.1–0.3% (w/w), and in particular with *Acropora digitifera*, for which the SOM concentration is about 0.2% of the skeleton weight. The influence of the ASM in the *in vitro* crystallization of calcium carbonate was tested in similar manner as the one used with other calcifying matrices in bulk [2], [74]–[76]. The *A. millepora* ASM has a strong effect on the shape of the crystals when compared with other species. For high concentrations of organic (10, 20 µg/mL) the morphologies of the produced crystals are drastically changed (completely round edges) but the nature of the polymorphs is calcite and vaterite throughout the experiment.

On gel, the ASM and the Laemmli-soluble fraction of the AIM are mainly polydisperse with only few discrete bands observed for the ASM, while Ca-binding experiments reveal few more bands that are not stained with silver nitrate. The SDS-PAGE pattern obtained with *A. millepora* resemble those obtained with the scleractinians *Stylophora pistillata* [15] and *A. digitifera* [73]. Polydispersity, the visualization of only a few discrete bands and the difficulty to stain gels with silver nitrate (‘negative staining’) are three common features encountered within skeletal matrices associated to calcium carbonate biominerals, and the skeletal matrix of *A. millepora* does not escape this rule. We assume that these drawbacks may be explained by the combination of few factors, among others, polyanionicity, glycosylation [77] and early degradation of the skeletal proteins.

One of the most striking findings on the biochemistry of *A. millepora* skeletal matrix concerns its sugar moieties. The remarkably high sugar content of the ASM is indeed confirmed by two techniques: FTIR and monosaccharide analysis after mild hydrolysis. In the first case, the FTIR spectra show that the amplitude of the sugar peaks is higher than that of proteins for the ASM and the AIM. In the second case, the analysis indicates that monosaccharides represent 52% (w/w) of the ASM. In that respect, this fraction exhibits a rather unusual biochemical signature: most of the data published so far on matrices associated to calcium carbonate biominerals in metazoans show that the sugar moieties, although essential from a functional viewpoint [78] are quantitatively minor in comparison to the protein moiety [78]–[80]. High amounts of monosaccharides may also be the reason why this matrix, when run on a monodimensional electrophoresis gel, stains poorly with silver nitrate and appears smeary. In comparison with the ASM, the sugar content of the AIM is ten times reduced (in %). However, its compositional pattern follows the same trend as the ASM; in particular, both matrices are extremely rich in arabinose, by far the dominant monosaccharide that represents about two thirds of the total monosaccharides in each of these fractions. To our knowledge, this signature is unique, since arabinose tends to be a minor monosaccharide in most of the calcium carbonate-associated matrices analyzed so far in mollusks [76], [78], [81]. These data are consistent – although more precise - with analyses previously published on the sugar contents of *Acropora* spp. from French Polynesia (ARA - 60.0%) and *Acropora danae* (ARA - 59.99%) [39]–[41]. This strongly suggests that a high content of arabinose in the ASM may be a biochemical trait of the genus *Acropora*. Even the scleractinian *Montipora*, the second richest in arabinose after *Acropora*, exhibits ‘only’ a percentage of this sugar slightly above 30% [39].

Moreover, it is interesting to notice that a high level of arabinose has also been detected in the liquid mucus released by *Acropora millepora* (63.2% mol of the total content in monosaccharides versus 65.8 and 68.8% mol in the ASM and AIM, respectively) [82]. By comparing the monosaccharide composition of the mucus and the SOM, the similarity observed between these two secretions is remarkable. Since the skeleton was thoroughly cleaned with sodium hypochlorite prior to any biochemical analysis, we can exclude with confidence that the analyzed ASM and AIM are contaminated by occluded mucus. We assert on the contrary that this similarity in terms of monosaccharide composition may reflect a true similarity in the glycosylation pattern of the two secretory products. In fact, this resemblance calls back to a previous finding showing that mucus and organic matrices have similar biochemical properties in mollusk and coral species [83]. At last, a brief overview in the literature indicates that arabinose-rich glycoproteins and polysaccharides are mainly found in plant cell walls, in particular in potato-lectins [84] and pectins [85], [86]. Data indicate that arabinose-rich pectins might play a role in intercellular attachment [85] and in the protection of cell walls of certain plants from desiccation [87]. Interestingly, at least three SOMPs were identified in the organic matrix of *A. millepora*, which contain lectin domains: MAM and fibronectin-containing protein and the SOM MAM and LDL-receptor 1 and 2 [3]. These proteins may have preference to bind arabinose, however in the present case, it is difficult to infer these functions without any further experimental characterization.

Another feature related to the saccharidic composition of the *A. millepora* skeletal matrix concerns the presence of chitin. In old studies, chitin – an insoluble polymer of N-acetyl-glucosamine and one key-macromolecule in several opisthokonts, in particular in arthropods and fungi - has been found to be an important constituent of the organic matrix of the scleractinian coral *Pocillopora damicornis* [45]. In our hands, glucosamine is a minor monosaccharide component of both ASM and AIM (7.3 and 7.2%, respectively) of *A. millepora* but it should not be excluded that a part of the glucosamine identified here may result from the conversion of the unstable N-acetyl-glucosamine residues during hydrolysis, and may consequently indicate the presence of chitin, in particular in the AIM. However, this will need to be tested further by crossing different analytical methods.

The proteomics investigations on the SOM of *A. millepora* indicate that the proteins are extremely diversified in terms of their primary structures, ranging from acidic to basic proteins. This finding challenges the classical molecular model of the skeletal matrix based almost exclusively on acidic proteins of the Asp-rich type [1]. We agree that Asp-rich proteins are essential players of the skeletal matrix. We believe that, from a quantitative viewpoint, they are dominant in the mixture of the skeletal matrix [3]. Previous amino-acid analyses on the bulk matrix of *Acropora* sp. have shown indeed that it is polyanionic and enriched in aspartic acid residues. Thus, the overall composition of the matrix may be dominated by SAARP 1 (20.4% Asp), SAARP 2 (21.1% Asp) and acidic SOMP (9.9% Asp) that exhibit pIs of 3.92, 4.13 and 4.24, respectively. However, these proteins are not solely in the matrix, the mucin-like proteins, coadhesin and MAM and LDL-receptors, all having extracellular matrix signatures in their structure, appear to be well-represented in the SOM based on their emPAI values [3]. In addition there are two other Asp-rich proteins, SAP1 and SAP2, and two proteins rich in glutamic acid: the Glu-rich protein (22.3%) and the USOMP6 (14.4%), though the Glu-rich protein has a very low pI (3.96) in contrast with USOMP6 (9.06).

In the recent proteomic study on the *Acropora* skeleton [3], there is evidence that some skeletal matrix proteins are initially membrane proteins, the extracellular part of which would be enzymatically cleaved and integrated in the matrix during the calcification process. This mechanism suggests the existence of a ‘physical’ molecular link at the interface between the calcifying epithelium and the end secretory product, the skeleton itself. Our assertion is supported not only by the presence of the transmembrane domains included in the SOMP primary structures but also by the fact that all the MS/MS matching peptides are located in the predicted extracellular parts of the identified proteins. Moreover, the sequences of the SOM proteins with transmembrane domains (TM) contain without exception cleavage sites on their extracellular side that may be cleaved by peptidases, which have been firmly identified as macromolecular components of the SOMPs. All these elements taken together strongly suggest that the extracellular domains of TM-containing proteins are cleaved enzymatically then occluded in the growing mineral phase during skeletogenesis.

In the present study, additional elements contribute to reassert this hypothesis. First of all, three of the acidic SOMPs (SAARP1, acidic SOMP 1, SAP1), as well as the hephaestin-like protein, contain predicted GPI-anchoring sites that enable these proteins to be anchored to the cell membrane facing the extracellular side [88]. Here too, these proteins are attached to the membrane before being subsequently released in the extracellular calcifying space.

Secondly, four SOMPs possess integrin-binding sites (3 residues-motifs) of the RGD type - Amil-Sap1, MAM and LDL-receptor domain-containing protein 1, collagen alpha-1 and PKD-1 related protein, while collagen alpha-1 chain, protocadherin-like and neuroglian-like proteins exhibit the ones of the NGR type [68]. Integrins are cell surface receptors that form a recognition system for cell migration, cell to extracellular matrix adhesion, and cell-to-cell adhesion [66], [68], when linked to proteins containing attachment sites such as RGD and NGR. Based on the presence of these integrin binding-sites, we can assume that the physical link between some of the skeletal matrix proteins and the cells of the calicoblastic epithelium is maintained in a certain way during the biomineralization process. This suggests that some SOMPs may be active in the subcalicoblastic space while remaining attached to the calicoblastic cells, until their release and subsequent occlusion in the growing skeleton. However, the exact mechanisms of adhesion and their effects on the biocalcification process would need to be tested more accurately.

## Supporting Information

Figure S1 **Spectra of the aragonitic skeleton with assignment of the main peaks. (A) FTIR. (B) Raman.**
(PDF)
