# Supplementary figures and images for: The Skeleton of the Staghorn Coral Acropora millepora: Molecular and Structural Characterization
Source: PLoS One. 2014 Jun 3;9(6):e97454. doi: 10.1371/journal.pone.0097454 (PMC4043741; doi:10.1371/journal.pone.0097454)

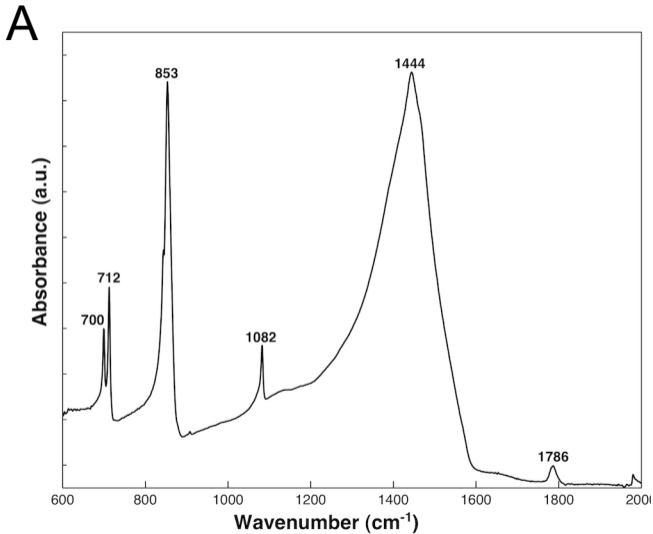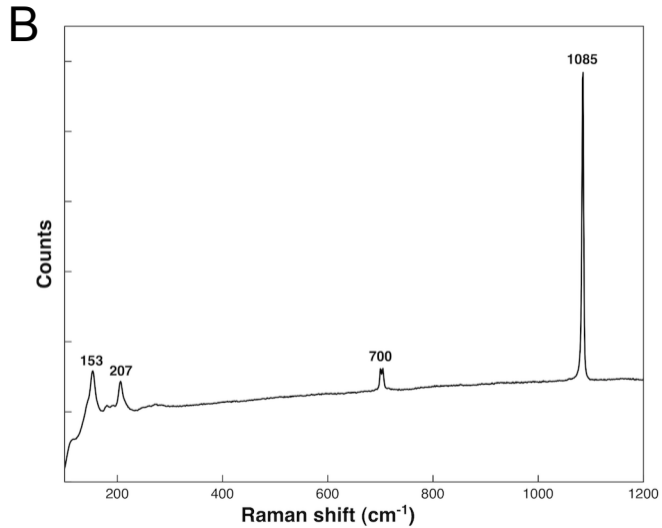

Supplement: Figure S1 — Spectra of the aragonitic skeleton with assignment of the main peaks. (A) FTIR. (B) Raman. (PDF) [file pone.0097454.s001.pdf]
